# Anchored phylogenomics illuminates the skipper butterfly tree of life

**DOI:** 10.1186/s12862-018-1216-z

**Published:** 2018-06-19

**Authors:** Emmanuel F. A. Toussaint, Jesse W. Breinholt, Chandra Earl, Andrew D. Warren, Andrew V. Z. Brower, Masaya Yago, Kelly M. Dexter, Marianne Espeland, Naomi E. Pierce, David J. Lohman, Akito Y. Kawahara

**Affiliations:** 1 Florida Museum of Natural History, University of Florida, Gainesville, Florida, 32611 USA; 2 RAPiD Genomics 747 SW 2nd Avenue IMB#14, Gainesville, FL 32601 USA; 3 Evolution and Ecology Group, Department of Biology, Middle Tennessee State University, Murfreesboro, TN 37132 USA; 4 The University Museum, The University of Tokyo, Hongo, Bunkyo-ku, Tokyo, 113-0033 Japan; 5 Arthropoda Department, Zoological Research Museum Alexander Koenig, Adenauer Allee 160, 53113 Bonn, Germany; 6 Department of Organismic and Evolutionary Biology, Harvard University, Cambridge, MA 02138 USA; 7 Biology Department, City College of New York, City University of New York, New York, NY 10031 USA; 8 Ph.D. Program in Biology, Graduate Center, City University of New York, New York, NY 10016 USA; 9 Entomology Section, National Museum of the Philippines, 1000 Manila, Philippines

**Keywords:** Anchored hybrid enrichment, Butterfly phylogenomics, Coalescent multi-species, Hesperiidae, Lepidoptera, Maximum likelihood, Molecular systematics, Papilionoidea, Parsimony, Target capture

## Abstract

**Background:**

Butterflies (Papilionoidea) are perhaps the most charismatic insect lineage, yet phylogenetic relationships among them remain incompletely studied and controversial. This is especially true for skippers (Hesperiidae), one of the most species-rich and poorly studied butterfly families.

**Methods:**

To infer a robust phylogenomic hypothesis for Hesperiidae, we sequenced nearly 400 loci using Anchored Hybrid Enrichment and sampled all tribes and more than 120 genera of skippers. Molecular datasets were analyzed using maximum-likelihood, parsimony and coalescent multi-species phylogenetic methods.

**Results:**

All analyses converged on a novel, robust phylogenetic hypothesis for skippers. Different optimality criteria and methodologies recovered almost identical phylogenetic trees with strong nodal support at nearly all nodes and all taxonomic levels. Our results support Coeliadinae as the sister group to the remaining skippers, the monotypic Euschemoninae as the sister group to all other subfamilies but Coeliadinae, and the monophyly of Eudaminae plus Pyrginae. Within Pyrginae, Celaenorrhinini and Tagiadini are sister groups, the Neotropical firetips, Pyrrhopygini, are sister to all other tribes but Celaenorrhinini and Tagiadini. Achlyodini is recovered as the sister group to Carcharodini, and Erynnini as sister group to Pyrgini. Within the grass skippers (Hesperiinae), there is strong support for the monophyly of Aeromachini plus remaining Hesperiinae. The giant skippers (*Agathymus* and *Megathymus*) once classified as a subfamily, are recovered as monophyletic with strong support, but are deeply nested within Hesperiinae.

**Conclusions:**

Anchored Hybrid Enrichment sequencing resulted in a large amount of data that built the foundation for a new, robust evolutionary tree of skippers. The newly inferred phylogenetic tree resolves long-standing systematic issues and changes our understanding of the skipper tree of life. These resultsenhance understanding of the evolution of one of the most species-rich butterfly families.

**Electronic supplementary material:**

The online version of this article (10.1186/s12862-018-1216-z) contains supplementary material, which is available to authorized users.

## Background

DNA sequencing and molecular phylogenetics have provided powerful tools to reconstruct the tree-of-life (ToL). Using a small number of gene fragments sampled across the genome, phylogenetic relationships of diverse groups have been inferred and their taxonomy revised. However, it has become evident that sampling such a small fraction of the genome often does not contain sufficient phylogenetic information to resolve long-standing systematic conundrums, or to allow inference of robust hypotheses of relationship for higher taxonomic groups. In the past decade, next-generation sequencing methods have stimulated the evolution of phylogenetics into phylogenomics, providing a means to generate data from hundreds or thousands of loci across the genome at relatively low cost. Target capture sequencing coupled with next-generation sequencing is a recently developed method for sampling large numbers of loci throughout the genome [1, 2]. Anchored Hybrid Enrichment (AHE [3]) is a form of target capture that captures moderately conserved loci by designing probes with dense tiling across loci in several lineages and enables the enrichment of orthologous loci from distantly related taxa. Genomic regions that hybridize with probes are enriched with PCR and can then be sequenced. AHE can work well for degraded DNA from older specimens [4], permitting the use of specimens with DNA too fragmented for PCR or reduced representation methods requiring restriction enzymatic cleavage. The use of AHE in systematics is still in its infancy, but pioneering studies have shown the astonishing potential of this method to fully resolve deeper parts of the ToL.

Trees constructed with AHE have clarified relationships among taxa that diverged long ago. Prum et al. [5] used AHE to sequence 259 loci and infer a novel and robust time-tree for birds, highlighting the power of the approach to shed light on ancient avian divergences that could not be resolved with 19 loci [6]. At a finer scale, Tucker et al. [7] resolved a long-standing debate regarding the classification of teiid lizards using AHE. Stout et al. [8] used AHE to infer the deeper-level phylogenetics of freshwater carps and loaches (Cypriniformes), while previous studies using a few loci inferred incongruent phylogenetic trees. Similarly, deep relationships across the jumping spider (Salticidae) tree-of-life were resolved using AHE [9]. Relationships within the angiosperm ToL that remained uncertain for decades have been clarified with AHE (e.g., sages [10]; sugarbushes [11]). A few studies have used AHE to address fine-scale evolutionary patterns within derived clades of the ToL.

Studies employing AHE have also resolved relationships among recently diverged lineages. Domingos et al. [12] used AHE to tackle species delimitation of *Tropidurus* lizards. Similarly, Ruane et al. [13] demonstrated that species trees estimated with a handful of loci were potentially biased and that an AHE approach allowed collection of sufficient sequence data to provide a robust phylogenetic estimate of pseudoxyrhophiine snakes. Anchored Hybrid Enrichment has also been shown useful for resolving different taxonomic levels across the spider ToL [14]. Early studies using AHE sampled relatively few taxa. Therefore, the utility of AHE to resolve both ancient and recent divergences in a single clade of the ToL has not been examined thoroughly [15].

With more than a million described species, insects are the most species-rich animal group on Earth, but only a few studies have examined their evolution using datasets including hundreds of loci sampled broadly across the genome. Young et al. [16] inferred a robust phylogeny of flower flies (Syrphidae) with AHE, and the nodal support of their tree was stronger than previous studies. Similarly, Winterton et al. [17] reconstructed a dated phylogeny of lacewings with an unprecedented level of resolution and robust nodal support. Haddad et al. [18] shed light on deep relationships of longhorn beetles (Cerambycidae) using an AHE dataset comprising 522 loci, providing the first robust phylogenetic hypothesis for the family. Using the same beetle AHE probe kit, Shin et al. [19] provided a much clearer picture of weevil phylogenetic relationships and evolutionary history. Breinholt et al. [20] used several AHE datasets to examine relationships across Lepidoptera and tested the utility of AHE to resolve relationships within superfamilies. This study corroborated the placement of butterflies as a derived moth clade, and demonstrated that strong nodal support can be achieved with AHE data. Recently, Espeland et al. [21] inferred a new phylogenomic framework for butterflies at the tribal level using an AHE approach, resolving the placement of clades that had been controversial for decades. The present study delves more deeply and examines relationships among butterflies at the genus-level.

Hesperiidae (or “skippers”) is a family of butterflies comprising more than 4200 described species [22], with highest diversity in the Neotropics. These fast-flying insects feed on angiosperms as larvae, are occasionally crepuscular, and are immediately recognized by their hook-shaped antennae, relatively chunky bodies, and characteristic silhouette while at rest. The family is cosmopolitan, and representatives can be found from the Arctic to the tropics, at nearly all elevations and in all terrestrial habitat types [23]. Unlike species in other butterfly families whose adults are frequently large and charismatic, skippers are often small and overlooked. Nevertheless, studies of their distribution, ecology and behavior have led to important discoveries. Skippers play a central role in pollination ecology. Recent studies have shown that species with long proboscides feed significantly more often on flowers with long nectar spurs [24] and are non-pollinating nectar thieves [25], affecting the pollination success of flowers with purloined nectar. However, in some angiosperm lineages such as orchids, skippers appear to be important pollinators [26]. Hesperiids have also been used as a model to study foraging strategies, demonstrating that switches between potential flower species for nectar occur in response to plant population density rather than specializing on particular flower species [27]. Competition for flower access between Neotropical hummingbirds and skippers has been suggested as one of the first examples of vertical partitioning of food resources in the context of interference competition [28]. Skippers have also been used as models in conservation and pest control pilot studies. A study of the silver-spotted skipper, *Hesperia comma*, in Britain showed that butterfly population decline could be arrested through adequate landscape-scale conservation policies coupled with favorable climatic change [29, 30]. During the end of the twentieth century, the banana skipper, *Erionota thrax*, had become a major pest across Southeast Asia and the Pacific and has been the subject of early pest management studies in Hawaii and New Guinea [31]. Skippers host plant use shows interesting ecological patterns that can be studied in an evolutionary context. For instance, Sahoo et al. [32] suggested that the evolution of monocot feeding in some lineages was associated with higher diversification rates. Dating of divergence times in butterflies suggests that skippers originated ca. 80 million years ago in the late Cretaceous ([21, 32, 33]).

Hesperiidae was historically thought to be the sister group to the remaining butterfly families, and has been treated as a suborder [34], a superfamily [35] or a family [36]. A century passed before molecular phylogenetic analyses seemed to substantiate skippers’ sister-relationship to all other butterflies (e.g., [37]). However, strong evidence now suggests that skippers are the sister-group to the “butterfly-moths”, Hedylidae, and are nested within the Papilionoidea [20, 21, 38–40].

**Fig. 1:**
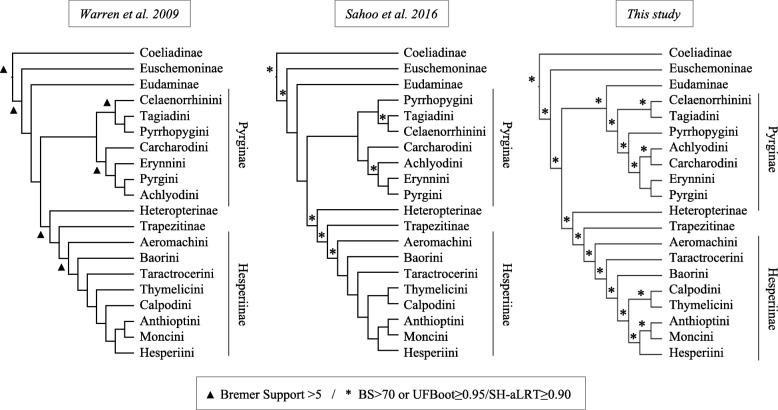
Summary of higher-taxonomy phylogenetic relationships of Hesperiidae . Phylogenetic trees representing the relationships among major groups of skippers as inferred by Warren et al. [23], Sahoo et al. [42] and the present study based on the nucleotide DT393 dataset with partitioning scheme selected in PartitionFinder and models of nucleotide substitution selected in IQ-TREE (Analysis A3). The topology of Sahoo et al. [32] is not shown because no nodal support values were reported in that paper. Two *incertae sedis* clades (see Fig. 3) are not shown

The first relatively comprehensive phylogenetic analyses of Hesperiidae were conducted only a decade ago by Warren et al. [23, 41]. Those results, which were based on three loci and morphological data for 196 genera, supported the monophyly of the family, and were used to revise subfamily and tribal boundaries. Sahoo et al. [32, 42] recently increased taxon and locus sampling to include 10 gene regions and approximately 300 species but, like Warren’s studies, did not recover strong support for most subfamilial and tribal-level relationships within the family (Fig. 1). For example, the placement of the monotypic Australian regent skipper *Euschemon rafflesia* (Euschemoninae) remained poorly supported (but see [43]). Similarly, the placement of the subfamily Eudaminae changed depending on the dataset and analysis employed [32, 42]. Finally, as found by Warren et al. [23, 41], the monophyly of Pyrginae and of some tribes within Hesperiinae was uncertain, with low nodal support for the placement of the different tribes (Fig. 1). These studies reveal the limits of phylogenetic inference using a small number of markers to infer higher-level relationships in rapidly diversifying taxa. A sufficiently large number of markers might help provide stronger support for the tree, but obtaining dozens or hundreds of homologous loci is impractical and expensive using traditional Sanger sequencing. Here, we implement a phylogenomic approach using AHE of hundreds of protein-coding loci to improve resolution of the skipper ToL and provide the first robust hypothesis for subfamilial and tribal relationships, on which patterns of diversification and biogeographic questions can be addressed.

## Methods

### Taxon sampling

We sampled 130 species from all recognized hesperiid subfamilies and tribes. Multiple exemplars from each tribe and genus were included for particularly species-rich lineages (Additional file 1**:** Table S1). We included three species of Hedylidae and one representative from each of the five other recognized butterfly families as outgroups (Additional file 1**:** Table S1). Specimens were collected from the wild and stored as (1) dried specimens in envelopes, (2) bodies preserved entirely in > 95% EtOH, or (3) legs kept in vial containing > 95% EtOH. For each specimen, the right-wing pair was preserved as a voucher, following a published protocol [44]. All extracts are stored at − 80 °C at the McGuire Center for Lepidoptera and Biodiversity (MGCL), Florida Museum of Natural History (Gainesville, USA).

### DNA extraction and Illumina sequencing

Genomic DNA was extracted using Omni Prep Genomic Extraction kits (G-Biosciences, Saint Louis, USA), which includes a chloroform phase separation step. Frozen, fresh or dried Lepidoptera tissue from the thorax or leg was homogenized in a 1:100 dilution of proteinase K to genomic lysis buffer in a centrifuge at 1200 rpm for 2.5 min at 25 °C. Samples were incubated overnight at 56 °C for approximately 12–18 h. The plate was then centrifuged at 3000 rpm for 3 min before 200 μL of chloroform was added to each sample. Samples were then centrifuged for 10 min at 4000 rpm to complete phase separation. Up to 80% of the supernatant was transferred to new, sterile 2.0 mL microtiter tubes and treated with 50 μL of DNA stripping buffer. Samples were incubated at 56 °C for 10 min, and tubes were cooled to room temperature before being centrifuged at 3000 rpm for 3 min before 100 μL of precipitation buffer was added to each sample along with 5 μL of mussel glycogen (10 mg/ml). Samples were mixed and centrifuged at 6000 rpm for 20 min to form a pellet. The supernatant was transferred to sterile 1.5 mL tubes and 500 μL of 2-propanol was added to each. Solutions were gently mixed and incubated at − 20 C for approximately 30 min before being centrifuged at 4000 rpm for 10 min. The DNA was cleaned by adding 700 μL ethanol and centrifuging at 4000 rpm for 10 min. Subsequently, the ethanol was removed, and DNA was eluted in 50 μL – 100 μL of TE buffer with the addition of 0.5 μL of RNase solution to remove traces of RNA. Samples were incubated overnight at 4 °C before DNA quantification. Some DNA extracts from the work of Warren et al. [41] were also included for AHE sequencing (see [41] for details of their DNA extraction protocol). DNA extracts were sent to RAPiD Genomics (Gainesville, FL, USA) for library preparation, hybrid enrichment, and sequencing. Libraries were constructed by mechanical shearing of DNA to an average size of 300 bp. Following shearing, end-repair reaction and ligation of an adenine residue to the 3′-end of the blunt-end fragments were performed to allow the ligation of barcoded adapters and PCR-amplification of the library. For AHE, a custom set of SureSelect probes (Agilent Technologies: Santa Clara, CA) were used for solution-based target enrichment of a pool containing 16 indexed libraries following the SureSelect Target Enrichment System for Illumina Paired-End Multiplexed Sequencing Library protocol. The Agilent Custom SureSelect kit used in this study (BUTTERFLY1.1 kit) is a modification (reduction from 425 to 391 loci with the best capture success across Papilionoidea and the addition of more reference sequences to improve capture) of the Papilionoidea-specific kit BUTTERFLY1.0 [21] which was derived by modifying and expanding the LEP1 probe set for Lepidoptera [20]. The BUTTERFLY1.1 kit loci names are consistent with loci names in BUTTERFLY1.0. This new kit includes 348 protein-coding loci that overlap with the AHE kit of Breinholt et al. [20].

### Data assembly

The data assembly pipeline largely followed Breinholt et al. [20]. TrimGalore! 0.4.0 (https://www.bioinformatics.babraham.ac.uk/projects/trim_galore/) was used to filter raw sequences with a minimum size of 30 bp. The assembly of each locus was performed using iterative baited assembly, with a Python script used to execute USEARCH [45] and Bridger [46] to assemble loci iteratively for each taxon. Orthology of sequences was assessed by using the single-hit and genome mapping location criteria defined by Breinholt et al. [20]. Single-hit and genome mapping location criteria uses NCBI BLASTN [47], with the genomic sequence of *Danaus plexippus* [48] as a reference, and scripts from Breinholt et al. [20] were applied to remove sequences for which the second-best hit had a bit score > 90% of the best hit bit score and confirming the location of the best hit to the orthologous loci. Contamination checks were conducted with USEARCH and Python scripts, to identify and remove nearly identical sequences across different subfamilies and genera. The final cleaned, assembled sequences trimmed to the probe region were aligned using MAFFT v. 7.245 [49] and strict consensus sequences for assembled isoforms were generated using FASconCAT-G 1.02 [50].

### Dataset construction

Individual gene fragment alignments were inspected and analyzed to remove stop codons, indels, sequencing errors and to assign correct protein-coding frames in AliView 1.18 [51]. To consider the potential impact of missing data, two datasets were generated: (1) a dataset comprising all loci that had been successfully captured, sequenced, and assembled from more than one taxon (total of 393 loci including two loci from BUTTERFLY1.0 that assembled although the probes were not included in BUTTERFLY1.1; this dataset is hereafter referred to as DT393); and (2) a more conservative dataset with fewer missing data, comprising all loci that had been successfully captured, sequenced and assembled for ≥70 taxa for a total of 369 loci, hereafter referred to as DT369. The two datasets were separately concatenated using FASconCAT-G 1.02. The raw individual locus alignments, cleaned concatenated alignments, and partitioning files used for phylogenetic inference in this study (see below) are available on Dryad (10.5061/dryad.bq10q98).

### Phylogenomic analyses

We inferred phylogenetic relationships under the maximum likelihood criterion using IQ-TREE 1.4.2 [52], in both unpartitioned and partitioned frameworks, to investigate the robustness of our inferences based on different partitioning schemes. We analyzed the dataset in three different ways: (A1) using a single concatenated alignment, (A2) using one partition per locus, and (A3) using a partitioning scheme determined in PartitionFinder 2.1.1 [53]. For the unpartitioned analyses A1 and the partitioned analyses (A2) (Table 1), the best models of nucleotide substitution were determined with the Bayesian Information criterion (BIC) using the command “TESTNEW” in IQ-TREE 1.4.2. For the partitioned analyses (A3), the optimal partitioning schemes were selected using the BIC in PartitionFinder 2.1.1. The search was based on a priori partitioning per locus with the rcluster algorithm [53] and the rcluster percentage set to 10 (default setting). Nucleotide substitution models for each partition were selected using BIC in IQ-TREE 1.4.2, across all available models including the FreeRate model (+R, [54]), which relaxes the assumption of gamma distributed rates. To assess nodal support, we performed 1000 ultrafast bootstrap replicates (UFBoot, [55]) with the “–bb” command. We also performed a SH-aLRT test [56] with 1000 replicates using the command “-alrt”. The UFBoot has been shown to be largely unbiased compared to standard or alternative bootstrap strategies, and SH-aLRT has been shown to be as conservative as standard bootstrap [55]. Only nodes with support values of UFBoot ≥ 95 and SH-aLRT ≥ 90 were considered robust.

**Table 1:** Results of the IQ-TREE maximum likelihood phylogenetic analyses

Analysis	Dataset	Data Type	Partitioning scheme	N. Partitions	Consensus LnL
*A1*	DT369	Nucleotides	None	1	−5115300.064
*A2*	DT369	Nucleotides	Locus	366	−5080057.834
*A3* ^a^	DT369	Nucleotides	PF	88	−5077543.916
*A5*	DT369	Amino Acids	None	1	−630708.956
*A6*	DT369	Amino Acids	Locus	366	−624687.178
*A7*	DT369	Amino Acids	PF	27	−627253.336
*A1*	DT393	Nucleotides	None	1	−5241440.350
*A2*	DT393	Nucleotides	Locus	393	−5204821.224
*A3* ^a^	DT393	Nucleotides	PF	94	−5203048.331
*A5*	DT393	Amino Acids	None	1	−662724.440
*A6*	DT393	Amino Acids	Locus	393	−656165.141
*A7*	DT393	Amino Acids	PF	28	−658908.662

*N. Partitions* number of partitions, *Consensus LnL* log-likelihood of the consensus of all bootstrap trees, *PF* PartitionFinder

^a^the two most likely scenarios for the two datasets

We also inferred species trees under the multi-species coalescent model in ASTRAL-II 4.10.0 [57]. Gene trees of each locus were estimated in IQ-TREE 1.4.2 for all 393 loci. Individual loci were not partitioned, and the best models of nucleotide substitution were selected using the BIC in IQ-TREE 1.4.2 across all available models. The maximum likelihood trees for each locus were assembled in two files, one for the 393 locus dataset and one for the 369 locus set and analyzed in ASTRAL-II 4.10.0 (Analyses A4). We performed 100 bootstrap replicates (ASTRALBoot) to assess the robustness of nodes in the final topologies, and calculated quartet scores (the number of gene tree quartets satisfied by the species tree) and normalized quartet scores (proportion of input gene tree quartet trees satisfied by the species tree). The higher the normalized quartet score, the less discordant individual gene trees are when compared to the species tree inferred in ASTRAL-II.

IQ-TREE and ASTRAL-II analyses were also conducted after translating the DT369 and DT393 nucleotide datasets into amino acids with FASconCAT-G 1.02. Unpartitioned analyses (A5) and analyses partitioned based on locus position (A6) (Table 1), were conducted as described above for the nucleotide analyses. Due to computational limitations, the optimal partitioning schemes for the partitioned analyses using PartitionFinder 2.1.1. (A7) were selected only based on the LG model, and the models of evolution were re-estimated using IQ-TREE 1.4.2 across all available models. The ASTRAL-II analyses based on amino acids (A8) were conducted as described above for nucleotide datasets. All analyses were performed on HiPerGator 2.0, the supercomputing cluster at the University of Florida (Gainesville, USA).

The amino acid and nucleotide DT393 datasets were also analyzed under the parsimony criterion in TNT 1.5 [58] (Analyses A9 and A10). The most parsimonious trees were inferred using a heuristic approach based on traditional tree searches, treating gaps as missing data and with all characters and transformations weighted equally. *Battus polydamas* was used as the outgroup in all TNT analyses. TNT searches were run with 200 replications of RAS (default setting) and branch-swapping operated with tree bisection-reconnection (TBR). Bremer support (BRS) values were calculated using 500 suboptimal Wagner trees that were 5000 steps longer on which TBR branch-swapping was operated.

## Results and discussion

At least 300 gene fragments were captured and sequenced from 136 of 138 species (98.6%), with an average of 350 loci per species (Additional files 1 and 2: Tables S1 and S2). Two loci from the BUTTERFLY1.0 kit that were not included in the BUTTERFLY1.1 kit were assembled and include in the final datasets (Table 2). Two species obtained via published transcriptomes had < 300 loci assembled; the skipper *Erynnis propertius* (Pyrginae, 155 loci; [59]), and the outgroup *Macrosoma hedylaria*, (Hedylidae, 175 loci; [40]). The capture success we obtained was remarkable, as skippers are often small, and in several cases, DNA was extracted from just two or three legs from of a single specimen. Our approach highlights the flexibility of AHE to generate genomic data for small specimens and/or small body parts using appropriate extraction protocols and library preparation.

**Table 2:** Results of the ASTRAL-II multispecies coalescent phylogenetic analyses

Analysis	Dataset	Data type	Quartet score	Normalized quartet score
*A4*	DT369	Nucleotides	3098963077	0.759
*A8*	DT369	Amino Acids	2035594777	0.501
*A4*	DT393	Nucleotides	3179504945	0.760
*A8*	DT393	Amino Acids	2082078892	0.501

**Fig. 2:**
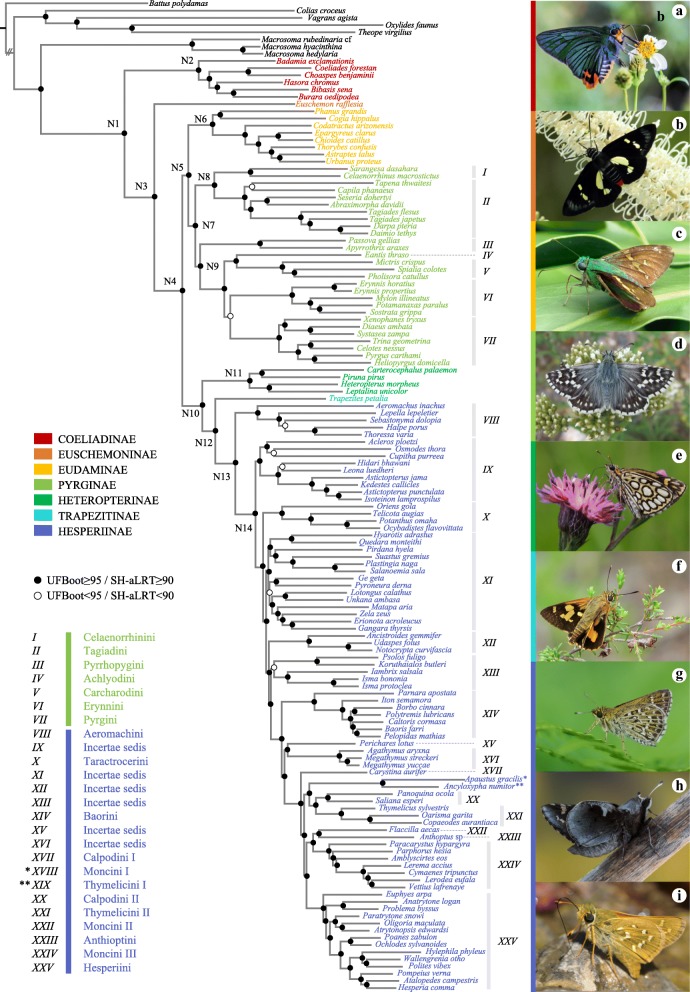
Phylogenomic skipper tree of life inferred using maximum likelihood . Maximum likelihood phylogeny inferred in IQ-TREE based on the nucleotide DT393 dataset with partitioning scheme selected in PartitionFinder and models of nucleotide substitution selected in IQ-TREE (Analysis A3). Nodal support values for numbered nodes on this tree (as well as alternative analyses) are presented in Fig. 3. Subfamilies and tribes recognized by Warren et al. [23] are indicated, and the color of species names indicates their subfamily. Images of skippers on the right illustrate morphological diversity within the family: **a** *Choaspes benjaminii* (credit: Sharleen Chao); **b** *Euschemon rafflesia* (credit: Todd Burrows); **c** *Astraptes talus* (credit: Les Catchick); **d** *Pyrgus carthami* (credit: Alan Cooper); **e** *Heteropterus morpheus* (credit: Hudák Tamás); **f** *Trapezites symmomus* (credit: John Tann); **g** *Aeromachus inachus* (credit: Tetsuya Shimizu); **h** *Megathymus yuccae* (credit: Jim & Lynne Weber); **i** *Hesperia comma* (credit: Pedro Candela)

**Fig. 3:**
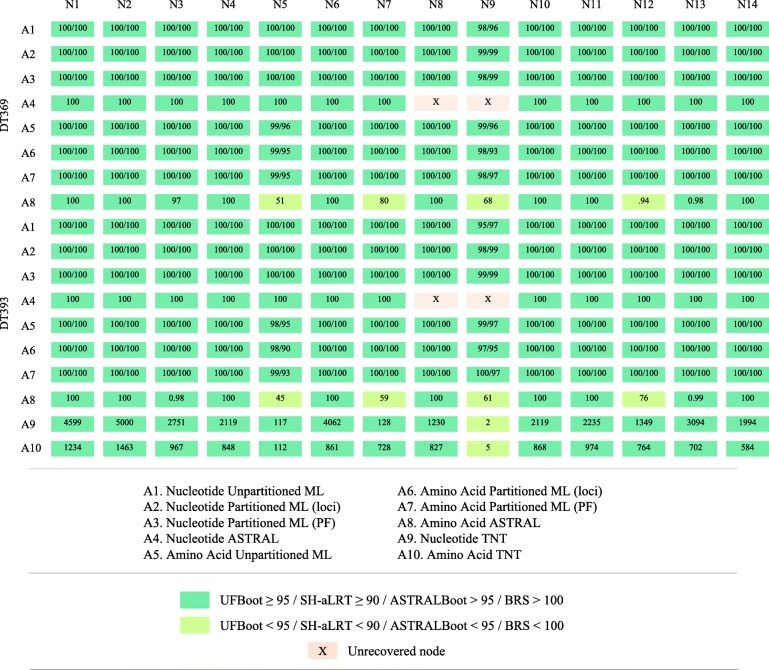
Nodal support along the backbone of the skipper tree of life . Summary of nodal support values for the deep divergences along the backbone of the skipper ToL inferred with IQ-TREE, ASTRAL-II and TNT phylogenetic analyses. Nodes N1 to N14 are indicated in Fig. 2. Alternative analyses A1 to A10 for both datasets (DT369 and DT393) are indicated in the embedded caption

Results of our phylogenomic analyses are summarized in Tables 1 and 2. Partitioning resulted in an improvement of the log-likelihood in IQ-TREE analyses, regardless of the dataset used (Table 1). IQ-TREE analyses with a partitioning scheme from PartitionFinder had the best log-likelihood for both DT369 and DT393 nucleotide datasets but not for the amino acid datasets. In Fig. 2, we present the IQ-TREE topology derived from the nucleotide dataset, DT393, based on the PartitionFinder partitioning scheme (Table 1). All phylogenetic analyses recovered a highly congruent, robust tree of all major, recognized deep-level divergences in Hesperiidae (Figs. 2 and 3). The TNT analyses of the amino acid DT393 dataset recovered four most parsimonious trees of 82,982 steps, while analyses of the nucleotide DT393 dataset recovered a unique most parsimonious tree of 1,266,723 steps. Nodal support values across the backbone were high in all analyses (see Fig. 2). The 14 backbone nodes of importance inferred using maximum likelihood had an UFBoot ≥95 and SH-aLRT ≥90, 10 out of 14 nodes had an ASTRALBoot ≥95, and 13 out of 14 nodes had a BRS > 100 (Figs. 2 and 3). ASTRAL-II and TNT analyses recovered a few nodes with ASTRALBoot < 95 or BRS < 5 among the most derived nodes of the backbone, especially in the subfamily Hesperiinae. The main discrepancies among analyses are restricted to relationships within a handful of derived clades, in which branching order varies slightly, depending on the dataset and methods.

Hesperiidae is recovered as monophyletic and sister to Hedylidae with strong nodal support (UFBoot = 100 / SH-aLRT = 100 / ASTRALBoot = 100 / BRS > 100) in all analyses, a result that is consistent with previous studies (e.g.*,* [20, 38]). Within Hesperiidae, all subfamilies recognized by Warren et al. [23] are recovered as monophyletic with strong nodal support (Figs. 2 and 3). The placement of *Euschemon* as sister to all skippers excluding Coeliadinae was suggested by Warren et al. [23, 41] and Zhang et al. [43], but contradicted by Sahoo et al. [32, 42] who recovered different topologies when using different analytical methods. Here we show that Coeliadinae is the sister-group to all remaining skippers, with the monotypic Euschemoninae as sister to all skippers excluding Coeliadinae (Figs. 2 and 3).

Another significant change within the skipper ToL is the placement of the Eudaminae (Figs. 1 and 2). Many prior studies placed Eudaminae as sister to all skippers except Coeliadinae and Euschemoninae ([23, 41, 42], see Fig. 1). Here, we recover Eudaminae as sister to the spread-winged skippers of the subfamily Pyrginae (Clades I–7 in Fig. 2) with robust nodal support in all analyses except the amino acid ASTRAL-II analyses (A8), which resulted in low nodal support (ASTRALBoot = 51, DT369; ASTRALBoot = 45, DT393). All but one eudamine genus (*Lobocla*) is found in the Neotropics, and the two early lineages Coeliadinae (from Africa to Oceania) and Euschemoninae (Australia) are distributed in the Old World, a result with important biogeographical implications. An Old World tropical origin of skippers is suggested by the current distribution of Coeliadinae and Euschemoninae.

Our prediction of the Old World tropical origin of Hesperiidae is supported to some extent by the fossil record. The oldest known butterfly fossil is in the Coeliadinae and was discovered on the island of Fur in Denmark, embedded in marine deposits dating back to the Eocene (ca. 55 Ma), thereby substantiating the existence of skippers in the Old-World during that period [60], and supporting the colonization of the New World from the Old World. Multiple origins of New World and Old World skipper lineages are evident across our tree, indicating a dynamic biogeographical history over time. However, the true number of colonization events, mechanisms involved (dispersal, regional extinction, vicariance), and the biogeographic history of skippers will require additional research with greater taxon sampling.

Within Pyrginae, we recovered relationships different from those of prior phylogenetic studies. Celaenorrhinini (Clade I) and Tagiadini (Clade II) are sister groups, as in Warren et al. [23, 41], Sahoo et al. [32, 42] and Espeland et al. [21]. However, the placement of the Neotropical firetips, Pyrrhopygini (Clade III), remains unclear. Most analyses recovered the tribe as sister to all Pyrginae except Celaenorrhinini and Tagiadini (Fig. 2), but with little support as in [19]. The ASTRAL-II nucleotide analyses recovered an alternative topology for the placement of Pyrrhopygini, as the sister group to Celaenorrhinini and Tagiadini with moderate support (ASTRALBoot = 73, DT369; ASTRALBoot = 85, DT393). This latter topological arrangement is consistent with results from previous studies [23, 32, 41, 42]. A greater and more diverse taxon sample could clarify the placement of firetips within Pyrginae. As in [19], we recovered Achlyodini (Clade IV) as sister to Carcharodini (Clade V), and Erynnini (Clade VI) as sister to Pyrgini (Clade VII), but with better support than in the previous study. These results are inconsistent with other previous studies that found alternative placements for these tribes within Pyrginae [23, 32, 42].

Our results also point to Heteropterinae as the sister-group to Trapezitinae + Hesperiinae, a relationship that is robust and congruent with previous work [21, 23, 32, 41–43]. The Australasian subfamily Trapezitinae, represented here by a single species of *Trapezites*, is recovered as sister to the grass skippers, Hesperiinae (Figs. 1, 3). This sister-group relationship is robust across analyses and congruent with prior studies [21, 23, 32, 41, 42]. Within Hesperiinae, relationships among tribes and clades are not fully resolved. We find Aeromachini (Clade VIII) as sister to the rest of Hesperiinae with strong nodal support. Finally, the giant skippers (*Agathymus* and *Megathymus* spp., Clade XVI) were recovered as monophyletic with strong nodal support in all analyses (Figs. 2 and 3). Most inferences (IQ-TREE and ASTRAL-II) based on nucleotide datasets placed them as sister to the Neotropical genus *Perichares*, although amino acid datasets placed them as an isolated clade within Hesperiinae. Based on morphology and the species’ known geographic distributions, the relationship of giant skippers with *Perichares* may be an artifact due to insufficient taxon sampling in this part of the tree and we do not consider this result to be robust. Several clades that remained unnamed by Warren et al. [23, 41] were also recovered in our analyses. We defer the proposal of new tribal names to a subsequent study with denser taxon sampling.

## Conclusions

The current study provides a robust evolutionary framework that was largely lacking for one of the most species-rich butterfly families. Nearly all relationships in our tree are strongly supported, regardless of the dataset type (amino acids or nucleotides), optimality criterion (likelihood, parsimony, coalescence), or partitioning scheme. Our study demonstrates that AHE is a robust method for inferring phylogenies over a range of taxonomic levels using different optimality criteria. The unprecedented amount of data generated from this study will permit reconstruction of intricate evolutionary patterns across the skipper tree of life, and shed light on new ones.

## Additional files

Additional file 1: Table of taxon sampling presenting detailed information for each specimen used in this study, along with number of captured AHE loci and genetic coverage in the AHE dataset. (XLSX 24 kb)

Additional file 2: Table presenting detailed information of the AHE matrix composition, including locus information, capture rate, probe region length, GC content and parsimony informative Sites. (XLSX 47 kb)
